# A posteriori error estimates of spectral method for nonlinear parabolic optimal control problem

**DOI:** 10.1186/s13660-018-1729-4

**Published:** 2018-06-19

**Authors:** Lin Li, Zuliang Lu, Wei Zhang, Fei Huang, Yin Yang

**Affiliations:** 1 Key Laboratory for Nonlinear Science and System Structure, Chongqing Three Gorges University, Chongqing, P.R. China; 2 Key Laboratory of Intelligent Information Processing and Control, Chongqing Three Gorges University, Chongqing, P.R. China; 3 Research Center for Mathematics and Economics, Tianjin University of Finance and Economics, Tianjin, P.R. China; 4 Key Laboratory of Intelligent Information Processing and Control of Chongqing Municipal Institutions of Higher Education, Chongqing Three Gorges University, Chongqing, P.R. China; 5 Hunan Key Laboratory for Computation and Simulation in Science and Engineering, Xiangtan University, Xiangtan, P.R. China; 6 Key Laboratory of Intelligent Computing and Information Processing of Ministry of Education, Xiangtan University, Xiangtan, P.R. China

**Keywords:** 49J20, 65N30, Optimal control problem, Nonlinear parabolic equations, Variational discretization, Spectral method, A posteriori error estimates

## Abstract

In this paper, we investigate the spectral approximation of optimal control problem governed by nonlinear parabolic equations. A spectral approximation scheme for the nonlinear parabolic optimal control problem is presented. We construct a fully discrete spectral approximation scheme by using the backward Euler scheme in time. Moreover, by using an orthogonal projection operator, we obtain $L^{2}(H^{1})-L^{2}(L ^{2})$ a posteriori error estimates of the approximation solutions for both the state and the control. Finally, by introducing two auxiliary equations, we also obtain $L^{2}(L^{2})-L^{2}(L^{2})$ a posteriori error estimates of the approximation solutions for both the state and the control.

## Introduction

Optimal control problems appear frequently in the operation of physical, social, economic processes, and other fields, and the numerical solutions of optimal control problems are extremely important for better performance of those fields. Therefore, one needs some efficient numerical methods to approximate the solutions of optimal control problems. Finite element methods seem to be the most popular used numerical methods in solving optimal control problems. Meanwhile, other numerical methods, such as the spectral method, the mixed finite element method and the finite volume method have also been applied to approximate some optimal control problems. For example, there has been done much work on the finite element method for optimal control problems [11, 13–15, 17], the spectral method for optimal control problems [4, 8, 9], the mixed finite element method for optimal control problems [3, 5–7, 18–20, 23, 24], and the finite volume method for optimal control problems [21, 22].

The spectral method has two important features: it enjoys the great superiority of fast convergence rate and provides very accurate approximations with a relatively small number of unknowns when the solutions are smooth. Let us briefly review the current literature. In [9], Ghanem and Sissaoui derived a posteriori error estimates by a spectral method of a linear–quadratic elliptic optimal control problem without inequality constraints. In [8], the authors studied the Legendre Galerkin spectral approximation of optimal control problems governed by elliptic equations and obtained a priori and a posteriori error estimates. A posteriori error estimates of a Legendre Galerkin spectral approximation of optimal control problems governed by parabolic equations were derived in [4]. To the best of our knowledge, a posteriori error estimates of the spectral method for nonlinear optimal control problems have never been studied. Nonlinear optimal control problems appear frequently in real life such as economics, chemical engineering, robotics and aeronautics, and the spectral method has several attractive features. Therefore, it is necessary to study a posteriori error estimates of the spectral method for nonlinear parabolic optimal control problems.

The purpose of this work is to derive a posteriori error estimates for the spectral approximation of an optimal control problem governed by nonlinear parabolic equations. We present a fully discrete scheme which uses the backward Euler scheme in time and uses the spectral approximation in space, and we obtain a posteriori error estimates of the spectral approximation solution for both the state and the control.

The outline of this paper is as follows. In Sect. 2, we shall construct spectral approximation scheme for nonlinear parabolic optimal control problem. In Sect. 3, by using orthogonal projection operator, $L^{2}(H^{1})-L^{2}(L^{2})$ a posteriori error estimates of the spectral approximation solutions for optimal control problem solution are derived. In Sect. 4, by using two auxiliary equations, $L^{2}(L^{2})-L ^{2}(L^{2})$ a posteriori error estimates of the spectral approximation solutions for optimal control problem are derived. In the last section, we briefly give conclusions and some possible future work.

Let Ω be bounded open sets in $\mathbb{R}^{2} $ with a Lipschitz boundary *∂*Ω. In this paper, we adopt the standard notation $W^{m,q}(\Omega )$ for Sobolev spaces on Ω with the norm $\Vert \cdot \Vert _{W^{m,q}(\Omega )}$ and the semi-norm $\vert \cdot \vert _{W ^{m,q}(\Omega )}$. We set $W_{0}^{m,q}(\Omega )\equiv \{w\in W^{m,q}( \Omega ): \partial^{\alpha }w|_{\partial \Omega } =0, \vert \alpha \vert \leq m-1\}$. We denote $W^{m,2}(\Omega )$ ($W_{0}^{m,2}(\Omega )$) by $H^{m}(\Omega )$ ($H_{0}^{m}(\Omega )$). We denote by $L^{s}(0,T;W ^{m,q}(\Omega ))$ the Banach space of all $L^{s}$ integrable functions from $(0,T)$ into $W^{m,q}(\Omega )$ with norm $\Vert v \Vert _{L^{s}(0,T;W ^{m,q}(\Omega ))}=(\int_{0}^{T} \Vert v \Vert ^{s}_{W^{m,q}(\Omega )}\,dt)^{ \frac{1}{s}}$ for $s\in [0,\infty )$ and the standard modification for $s=\infty $. Similarly, we define the spaces $H^{1}(0,T;W^{m,q}( \Omega ))$ and $C^{l}(0,T;W^{m,q}(\Omega ))$, the details can be found in [12].

## Spectral approximation of nonlinear parabolic optimal control

In this section, we shall state the spectral approximation scheme and its optimality conditions for the optimal control problem governed by nonlinear parabolic equations. Now, we set the state space $W=L^{2}(0,T;H _{0}^{1}(\Omega ))$, the control space $X=L^{2}(0,T;L^{2}(\Omega ))$, and $V=H_{0}^{1}(\Omega )$. We will study the following nonlinear parabolic optimal control problem:

2.1 $$\begin{aligned}& \min_{u\in X; u(t)\in K} \biggl\{ \frac{1}{2} \int_{0}^{T}\bigl( \Vert y-y_{d} \Vert ^{2}_{L^{2}(\Omega )}+ \Vert u \Vert ^{2}_{L^{2}( \Omega )} \bigr)\,dt \biggr\} , \end{aligned}$$

2.2 $$\begin{aligned}& y_{t}- \operatorname{div} (A\nabla y)+\phi (y)=f+Bu, \quad x\in \Omega ,t\in (0,T], \end{aligned}$$

2.3 $$\begin{aligned}& y| _{\partial \Omega }=0, \quad t\in [0,T], \end{aligned}$$

2.4 $$\begin{aligned}& y(x,0)=y_{0}(x), \quad x\in \Omega , \end{aligned}$$

where *K* is a set defined by

$$ K= \biggl\{ v\in X: \int_{0}^{T} \int_{\Omega }v\,dx\,dt\geq 0 \biggr\} , $$

and $f,y_{d}\in L^{2}(0,T;L^{2}(\Omega ))$, $y_{0}\in H_{0}^{1}( \Omega )$, let *B* be a linear continuous operator from *X* to $L^{2}(0,T;V^{\prime })$, $\phi (\cdot )\in W^{2,\infty }(-R,-R)$ for any $R>0$, $\phi '(y)\in L^{2}(\Omega )$ for any $y\in H^{1}(\Omega )$, $\phi '\geq 0$, and the matrix $A(\cdot )=(a_{i,j}(\cdot ))_{2\times 2}\in (C^{\infty }(\bar{\Omega }))^{2\times 2}$, such that there is a constant $c>0$ satisfying

$$ \xi^{t}A\xi \geq c \Vert \xi \Vert ^{2}, \quad \xi \in \mathbb{R}^{2}. $$

Let

$$\begin{aligned}& a(y,w)= \int_{\Omega }(A\nabla y)\cdot \nabla w \,dx, \quad \forall\ y,w \in H_{0}^{1}(\Omega ), \\& (f_{1},f_{2})= \int_{\Omega } f_{1}f_{2}\,dx, \quad \forall\ f_{1},f_{2} \in L^{2}(\Omega ), \\& (u,v)= \int_{\Omega }uv\,dx, \quad \forall\ u,v\in L^{2}(\Omega ). \end{aligned}$$

It is well known that there are constants *c* and $C>0$ such that

$$ a(v,v)\geq c \Vert v \Vert _{1, \Omega }^{2},\qquad \bigl\vert a(v,w) \bigr\vert \leq C \vert v \vert _{1, \Omega } \vert w \vert _{1, \Omega }, \quad \forall\ v,w\in H_{0}^{1}(\Omega ). $$

Then a weak formula of the optimal control problem can be obtained:

2.5 $$ \min_{u(t)\in K} \biggl\{ \frac{1}{2} \int_{0}^{T}\bigl( \Vert y-y_{d} \Vert ^{2}_{L ^{2}(\Omega )}+ \Vert u \Vert ^{2}_{L^{2}(\Omega )} \bigr)\,dt \biggr\} , $$

where $y\in W$, $u\in X$, $u(t)\in K$ subject to

2.6 $$\begin{aligned}& (y_{t},w)+ a(y,w)+\bigl(\phi (y),w\bigr)=(f+Bu,w), \quad \forall\ w \in V, t\in (0,T], \end{aligned}$$

2.7 $$\begin{aligned}& y(x,0)=y_{0}(x), \quad x\in \Omega . \end{aligned}$$

It is well known (see, e.g., [16]) that the optimal control problem (2.5)–(2.7) has at least one solution $(y,p,u)$, and that if a triplet $(y,p,u)$ is the solution of (2.5)–(2.7), then there is a co-state $p\in W$ such that $(y,p,u)$ satisfies the following optimality conditions:

2.8 $$\begin{aligned}& (y_{t},w)+ a(y,w)+\bigl(\phi (y),w\bigr)=(f+Bu,w), \quad \forall\ w \in V, y(0)=y_{0}, \end{aligned}$$

2.9 $$\begin{aligned}& -(p_{t},q)+ a(q,p)+\bigl(\phi '(y)p,q \bigr)=(y-y_{d}, q), \quad \forall\ q \in V, p(T)=0, \end{aligned}$$

2.10 $$\begin{aligned}& \int_{0}^{T}\bigl(u+B^{*}p, v-u\bigr) \,dt \geq 0, \quad \forall\ v(t) \in K, v\in X, \end{aligned}$$

where $B^{*}$ is the adjoint operator of *B*.

### Lemma 2.1

*Let* $(y,p,u)$ *be the solution of* (2.8)*–*(2.10), *we have*

$$ u=\max\bigl\{ 0,\overline{B^{*}p}\bigr\} -B^{*}p, $$

*where*

$$ \overline{B^{*}p}=\frac{\int_{0}^{T}\int_{\Omega }B^{*}p\,dx\,dt}{\int _{0}^{T}\int_{\Omega }1\,dx\,dt} $$

*denotes the integral average on* $\Omega \times [0,T]$ *of the function* $B^{*}p$.

### Proof

For any function $p\in W$, we have

$$ u=\max\bigl\{ 0,\overline{B^{*}p}\bigr\} -B^{*}p, $$

which satisfies the following variational inequality:

$$ \int_{0}^{T}\bigl(u+B^{*}p, v-u\bigr) \,dt \geq 0, \quad \forall\ v(t) \in K. $$

If $\overline{B^{*}p}>0$, then it is easy to see $u=\overline{B^{*}p}-B ^{*}p$ and

$$\begin{aligned} \int_{0}^{T}\bigl(u+B^{*}p, v-u\bigr) \,dt &= \int_{0}^{T} \int_{\Omega }\bigl(u+B^{*}p\bigr) ( v-u)\,dx\,dt \\ &= \int_{0}^{T} \int_{\Omega }\overline{B^{*}p}\bigl( v- \overline{B^{*}p}+B ^{*}p\bigr)\,dx\,dt \\ &=\overline{B^{*}p} \int_{0}^{T} \int_{\Omega }v \,dx\,dt\geq 0, \quad \forall\ v(t) \in K. \end{aligned}$$

If $\overline{B^{*}p}\leq 0$, then it is clear that $u=-B^{*}p$ and

$$ \int_{0}^{T}\bigl(u+B^{*}p, v-u\bigr) \,dt \geq 0. $$

So we have $u=\max\{0,\overline{B^{*}p}\}-B^{*}p$. □

Next, we will use the Legendre Galerkin spectral method to investigate the spectral approximation of the nonlinear parabolic optimal control problem (2.8)–(2.10). From now on, we assume that $\Omega =(-1,1)^{2}$. Firstly, let us introduce some basic notations which will be used in the sequel. For $x_{i}$, $i=1,2$, we denote by $L_{r}(x_{i})$ the *r*th degree Legendre polynomial in the variable $x_{i}$, and we set

$$ X^{i}_{N}=\operatorname{span}\bigl\{ L_{0}(x_{i}), L_{1}(x_{i}),\ldots, L _{N}(x_{i}) \bigr\} , $$

where $N\geq 0$ is an integer. We define a product space such as

$$ X_{N}=\prod_{i=1}^{2} X_{N}^{i}. $$

We introduce the finite dimensional spaces $V^{N}=X_{N}\bigcap V$ and $K^{N}=X_{N} \bigcap K$. In addition, *C* denotes a general positive constant independent of *N*, the order of the spectral approximation.

Then the Legendre Galerkin spectral approximation for the nonlinear parabolic optimal control problem is

2.11 $$\begin{aligned}& \min_{u_{N}(t)\in K^{N}} \biggl\{ \frac{1}{2} \int_{0}^{T}\bigl( \Vert y_{N}-y _{d} \Vert ^{2}_{L^{2}(\Omega )}+ \Vert u_{N} \Vert ^{2}_{L^{2}(\Omega )}\bigr)\,dt \biggr\} , \end{aligned}$$

2.12 $$\begin{aligned}& (y_{Nt},w_{N})+ a(y_{N},w_{N})+ \bigl(\phi (y_{N}),w_{N}\bigr)=(f+Bu_{N},w_{N}), \quad \forall\ w_{N} \in V^{N}, \end{aligned}$$

2.13 $$\begin{aligned}& y_{N}(x,0)=y_{0}^{N}(x), \quad x\in \Omega , \end{aligned}$$

where $y_{N}\in H^{1}(0,T;V^{N})$ and $y_{0}^{N}\in V^{N}$ is an approximation of $y_{0}$.

It follows that the optimal control problem (2.11)–(2.13) has at least one solution $(y_{N},u_{N})$, and that if a pair $(y_{N},u_{N})$ is the solution of (2.11)–(2.13), then there is a co-state $p_{N}$ such that $(y_{N},p_{N} ,u_{N})$ satisfies the following optimality conditions:

2.14 $$\begin{aligned}& (y_{Nt},w_{N})+a(y_{N},w_{N})+ \bigl(\phi (y_{N}),w_{N}\bigr)=(f+Bu_{N},w_{N}), \quad \forall\ w_{N} \in V^{N}, \end{aligned}$$

2.15 $$\begin{aligned}& y_{N}(x,0)=y_{0}^{N}(x), \quad x\in \Omega , \\& {} -(p_{Nt},q_{N})+a(q_{N},p_{N})+ \bigl(\phi '(y_{N})p_{N},q_{N} \bigr)=(y_{N}-y _{d},q_{N}), \quad \forall\ q_{N} \in V^{N}, \end{aligned}$$

2.16 $$\begin{aligned}& p_{N}(x,T)=0, \quad x\in \Omega , \\& \bigl(u_{N}+B^{*}p_{N}, v_{N}-u_{N}\bigr)\geq 0, \quad u_{N}(t)\in K^{N}, \forall\ v_{N}\in K^{N}. \end{aligned}$$

Now, we shall construct the fully discrete approximation scheme for the above semi-discrete problem. Let $0=t_{0}< t_{1}<\cdots <t_{M-1}<t_{M}=T$, $k_{i}=t_{i}-t_{i-1}$, $i=1,2,\ldots,M$, $k=\max_{1\leq i\leq N} \{k_{i}\}$. For $i=1,2,\ldots,M$, construct the approximation spaces $V_{i}^{N}\subset H_{0}^{1}(\Omega )$ (similar as $V^{N}$) on the *i*th time step. Similarly, construct the approximation spaces $K_{i}^{N}\subset L^{2}(\Omega )$ (similar to $K^{N}$) on the *i*th time step. The fully discrete approximation scheme of (2.11)–(2.13) is to find $(y_{N}^{i},u_{N}^{i})\in V_{i} ^{N}\times K_{i}^{N}$, $i=1,2,\ldots,M$, such that

2.17 $$\begin{aligned}& \min_{u_{N}^{i}\in K_{i}^{N}} \Biggl\{ \frac{1}{2}\sum _{i=1}^{N}k _{i} \bigl( \bigl\Vert y_{N}^{i}-y_{d}(x,t_{i}) \bigr\Vert ^{2}_{L^{2}( \Omega )}+ \bigl\Vert u_{N}^{i} \bigr\Vert ^{2}_{L^{2}(\Omega )} \bigr) \Biggr\} , \end{aligned}$$

2.18 $$\begin{aligned}& \begin{aligned}[b] &\biggl( \frac{y_{N}^{i}-y_{N}^{i-1}}{k_{i}},w_{N} \biggr) +a \bigl(y_{N}^{i},w _{N}\bigr)+\bigl(\phi \bigl(y^{i}_{N}\bigr),w_{N}\bigr)= \bigl(f(x,t_{i})+ Bu_{N}^{i},w_{N} \bigr), \\ &\quad \forall\ w_{N}\in V_{i}^{N}, i=1,2,\ldots,M, \end{aligned} \end{aligned}$$

2.19 $$\begin{aligned}& y_{N}^{0}(x)=y_{0}^{N}(x), \quad x\in \Omega . \end{aligned}$$

It follows that the optimal control problem (2.17)–(2.19) has at least one solution $(Y_{N}^{i},U_{N}^{i})$, $i=1,2,\ldots,M$, and that if a pair $(Y_{N}^{i},U_{N}^{i})\in V_{i}^{N}\times K_{i}^{N}$, $i=1,2,\ldots,M$, is the solution of (2.17)–(2.19), then there is a co-state $P_{N}^{i-1}\in V_{i}^{N}$, $i=M,\ldots,2,1$, such that the triplet $(Y_{N}^{i},P_{N}^{i-1},U_{N}^{i})\in V_{i}^{N}\times V _{i}^{N}\times K_{i}^{N}$, $i=1,2,\ldots,M$, satisfies the following optimality conditions:

2.20 $$\begin{aligned}& \begin{aligned}[b] & \biggl( \frac{Y_{N}^{i}-Y_{N}^{i-1}}{k_{i}},w_{N} \biggr) +a \bigl(Y_{N} ^{i},w_{N}\bigr)+\bigl(\phi \bigl(Y_{N}^{i}\bigr),w_{N}\bigr)= \bigl(f(x,t_{i})+ BU_{N}^{i},w_{N} \bigr), \\ & \quad \forall\ w_{N}\in V_{i}^{N}, i=1,2,\ldots,M, \end{aligned} \end{aligned}$$

2.21 $$\begin{aligned}& Y_{N}^{0}(x)=y_{0}^{N}(x),\quad x \in \Omega , \end{aligned}$$

2.22 $$\begin{aligned}& \begin{aligned}[b]& \biggl( \frac{P_{N}^{i-1}-P_{N}^{i}}{k_{i}},q_{N} \biggr) +a \bigl(q_{N},P _{N}^{i-1}\bigr)+\bigl(\phi '\bigl(Y_{N}^{i-1}\bigr)p_{N}^{i-1},q_{N} \bigr)=\bigl(Y_{N}^{i}-y_{d}(x,t _{i}),q_{N}\bigr), \\ & \quad \forall\ q_{N}\in V_{i}^{N}, i=M,\ldots,2,1, \end{aligned} \end{aligned}$$

2.23 $$\begin{aligned}& P_{N}^{M}(x) = 0,\quad x\in \Omega , \end{aligned}$$

2.24 $$\begin{aligned}& \bigl(U_{N}^{i}+B^{*}P_{N}^{i-1}, v_{N}-U_{N}^{i}\bigr)\geq 0, \quad \forall\ v_{N}\in K_{i}^{N}, i=1,2,\ldots,M. \end{aligned}$$

For $i=1,2,\ldots,M$, let

$$\begin{aligned}& Y_{N} | _{(t_{i-1},t_{i}]}=\bigl((t_{i}-t) Y_{N}^{i-1}+(t-t_{i-1})Y_{N} ^{i}\bigr)/k_{i}, \\& P_{N} | _{(t_{i-1},t_{i}]}=\bigl((t_{i}-t) P_{N}^{i-1}+(t-t_{i-1})P_{N} ^{i}\bigr)/k_{i}, \\& U_{N}| _{(t_{i-1},t_{i}]}=U_{N}^{i}. \end{aligned}$$

For any function $w\in C(0,T;L^{2}(\Omega ))$, let $\hat{w}(x,t)| _{t \in (t_{i-1},t_{i}] }= w(x,t_{i})$, $\tilde{w}(x,t)| _{t\in (t_{i-1},t _{i}]}=w(x,t_{i-1})$. Then the optimality conditions (2.20)–(2.24) can be restated:

2.25 $$\begin{aligned}& \begin{aligned}[b]& (Y_{Nt},w_{N})+a(\hat{Y}_{N},w_{N})+ \bigl(\phi (\hat{Y}_{N}),w_{N}\bigr)=( \hat{f}+BU_{N},w_{N}), \\ & \quad \forall\ w_{N}\in V_{i}^{N}, t\in (t_{i-1},t_{i}], i=1,2,\ldots,M, \end{aligned} \end{aligned}$$

2.26 $$\begin{aligned}& Y_{N}(x,0) = y_{0}^{N}(x),\quad x\in \Omega , \end{aligned}$$

2.27 $$\begin{aligned}& \begin{aligned}[b]& {} -(P_{Nt},q_{N})+a(q_{N}, \tilde{P}_{N})+\bigl(\phi '(\tilde{Y}_{N}) \tilde{P}_{N},q_{N}\bigr)=(\hat{Y}_{N}- \hat{y}_{d},q_{N}), \\ & \quad \forall\ q_{N}\in V_{i}^{N}, t\in (t_{i-1},t_{i}], i=M,\ldots,2,1, \end{aligned} \end{aligned}$$

2.28 $$\begin{aligned}& P_{N}(x,T) = 0,\quad x\in \Omega , \end{aligned}$$

2.29 $$\begin{aligned}& \begin{aligned}[b]& \bigl(U_{N}+B^{*}\tilde{P}_{N}, v_{N}-U_{N}\bigr)\geq 0,\quad U_{N}\in K_{i} ^{N}, \\ & \quad \forall\ v_{N}\in K_{i}^{N}, t\in (t_{i-1},t_{i}], i=1,2,\ldots,M. \end{aligned} \end{aligned}$$

For any $u \in L^{2}(\Omega )$, we define the orthogonal projection operator $P_{N}: L^{2}(\Omega )\rightarrow K^{N} $ which satisfies

$$ (P_{N}u-u, w_{N})=0, \quad \forall\ w_{N} \in K^{N}. $$

It can be shown (see [2]) that

$$ P_{N}u=\sum_{\max _{1\leq i \leq 2} \{k_{i}\} \leq N} \hat{u}_{k} \phi_{k}, \quad \hat{u}_{k}=\prod _{i=1}^{2} \biggl( k _{i}+\frac{1}{2} \biggr) \int_{\Omega }u(x)\phi_{k}(x)\,dx, $$

where $k=(k_{1},k_{2})$ and $\phi_{k}(x)=L_{k_{1}}(x_{1}) L_{k_{2}}(x _{2})$.

For any $u \in H_{0}^{1}(\Omega )$, $P_{1,N}^{0} :H_{0}^{1}(\Omega )\rightarrow V^{N}$ is defined by

$$ \int_{\Omega }\nabla \bigl(u-P_{1,N}^{0}u\bigr) \cdot \nabla w\,dx =0, \quad \forall\ w\in V^{N}. $$

The following lemma will play a very important role in a posteriori error estimates. It can be found in the reference book of Ref. [2].

### Lemma 2.2

*For all* $u\in H^{m}(\Omega )$ ($m\geq 0$), *we have*

$$ \Vert u-P_{N}u \Vert _{H^{l}(\Omega )}\leq CN^{\sigma (l)-m} \Vert u \Vert _{H^{m}( \Omega )}, \quad 0\leq l \leq m, $$

*where* $\sigma (l)=0$ *if* $l=0$, *and* $\sigma (l)=2l-\frac{1}{2}$ *for* $l>0$. *If* $u\in H_{0}^{1}(\Omega )\cap H^{m}(\Omega )$, $m \geq 1$, *then we have*

$$ \bigl\Vert u-P_{1,N}^{0}u \bigr\Vert _{H^{\mu }(\Omega )} \leq C N^{\mu -m} \Vert u \Vert _{H^{m}(\Omega )}, \quad 0\leq \mu \leq 1. $$

For $\varphi \in V^{N}$, we shall write

2.30 $$\begin{aligned}& \phi (\varphi )-\phi (\rho )=-\tilde{\phi }^{\prime }( \varphi ) ( \rho -\varphi )=-\phi^{\prime }(\rho ) (\rho -\varphi ) +\tilde{ \phi } ^{\prime \prime }(\varphi ) (\rho -\varphi )^{2}, \end{aligned}$$

where

$$ \tilde{\phi }^{\prime }(\varphi )= \int_{0}^{1}\phi^{\prime }\bigl(\varphi +s(\rho -\varphi )\bigr)\,ds,\quad\quad \tilde{\phi }^{\prime \prime }(\varphi )= \int_{0}^{1}(1-s)\phi^{\prime \prime }\bigl(\rho +s( \varphi -\rho )\bigr)\,ds $$

are bounded functions in Ω̄, more details can be found in [7].

## $L^{2}(H^{1})-L^{2}(L^{2})$ a posteriori error estimates

In this section, we shall derive a $L^{2}(H^{1})-L^{2}(L^{2})$ posteriori error estimates for the spectral approximation of the optimal control problem governed by nonlinear parabolic equations. Set

$$\begin{aligned}& J(u)=\frac{1}{2} \int_{0}^{T} \bigl( \Vert y-y_{d} \Vert ^{2}_{L^{2}(\Omega )}+ \Vert u \Vert ^{2}_{L^{2}(\Omega )} \bigr) \,dt, \\& J_{N}(U_{N})=\frac{1}{2} \int_{0}^{T} \bigl( \Vert Y_{N}-y_{d} \Vert ^{2} _{L^{2}(\Omega )}+ \Vert U_{N} \Vert ^{2}_{L^{2}(\Omega )} \bigr) \,dt. \end{aligned}$$

It can be shown that (see [12])

3.1 $$\begin{aligned}& \bigl(J^{\prime }(u),v\bigr)=\bigl(u+B^{*}p,v \bigr), \end{aligned}$$

3.2 $$\begin{aligned}& \bigl(J_{N}^{\prime }(U_{N}),v\bigr)= \bigl(U_{N}+B^{*}\tilde{P}_{N},v\bigr), \end{aligned}$$

3.3 $$\begin{aligned}& \bigl(J^{\prime }(U_{N}),v\bigr)= \bigl(U_{N}+B^{*}p(U_{N}),v\bigr), \end{aligned}$$

where $p(U_{N})$ is the solution of the auxiliary equations:

3.4 $$\begin{aligned}& \bigl(y_{t}(U_{N}),w\bigr)+a \bigl(y(U_{N}),w\bigr)+\bigl(\phi \bigl(y(U_{N})\bigr),w \bigr)=(f+BU_{N},w), \quad \forall\ w\in V, \end{aligned}$$

3.5 $$\begin{aligned}& y(U_{N}) (x,0)=y_{0}(x), \quad x\in \Omega , \end{aligned}$$

3.6 $$\begin{aligned}& \begin{aligned}[b]& {} -\bigl(p_{t}(U_{N} ),q\bigr)+a \bigl(q,p(U_{N})\bigr)+\bigl(\phi '\bigl(y(U_{N}) \bigr)p(U_{N}),q\bigr) \\ &\quad =\bigl(y(U_{N}) -y_{d},q \bigr), \quad \forall\ q\in V,\end{aligned} \end{aligned}$$

3.7 $$\begin{aligned}& p(U_{N}) (x,T)=0, \quad x\in \Omega . \end{aligned}$$

We assume that the cost function *J* is strictly convex near the solution *u*, i.e., for the solution *u* there exists a neighborhood of *u* in $L^{2}$ such that *J* is convex in the sense that there is a constant $c>0$ satisfying

3.8 $$\begin{aligned}& \bigl(J^{\prime }(u)-J^{\prime }(v), u-v\bigr)\geq c \Vert u-v \Vert ^{2}, \end{aligned}$$

for all *v* in this neighborhood of *u*. The convexity of $J(\cdot )$ is closely related to the second order sufficient optimality conditions of optimal control problems, which are assumed in many studies on numerical methods of the problem. For instance, in many references, the authors assume the following second order sufficiently optimality condition (see [13]): there is $c >0$ such that $J^{\prime \prime }(u)v^{2}\geq c \Vert v \Vert _{0}^{2}$.

From the assumption (3.8), by the proof contained in [1], there is a $c>0$ independent of *N*, such that

3.9 $$ \int_{0}^{T}\bigl(J'(u)-J'(u_{N}),u-u_{N} \bigr)\,dt\geq c \Vert u-u_{N} \Vert _{L^{2}(0,T;L ^{2}(\Omega ))}^{2}, $$

where *u* and $u_{N}$ are the solutions of (2.8)–(2.10) and (2.14)–(2.16), respectively.

### Theorem 3.1

*Let* $(y,p,u)$ *and* $(Y_{N},P_{N},U_{N})$ *be the solutions of* (2.8)*–*(2.10) *and* (2.25)*–*(2.29), *respectively*. *Then we have*

3.10 $$ \Vert u-U_{N} \Vert ^{2}_{L^{2}(0,T;L^{2}(\Omega ))} \leq C\eta_{1}^{2}+C \bigl\Vert p(U _{N})- \tilde{P}_{N} \bigr\Vert ^{2}_{L^{2}(0,T;L^{2}(\Omega ))}, $$

*where* $p(U_{N})$ *is defined by* (3.5) *and*

$$ \eta_{1}^{2}=\sum_{i=1}^{M} \int_{t_{i-1}}^{t_{i}} \bigl\Vert U_{N}+B^{*} \tilde{P}_{N} \bigr\Vert _{L^{2}(\Omega )}^{2}\,dt. $$

### Proof

According to (3.9), we have

$$\begin{aligned} c \Vert u-U_{N} \Vert ^{2}_{L^{2}(0,T;L^{2}(\Omega ))} &\leq \int_{0}^{T}\bigl(J'(u)-J'(U _{N}), u-U_{N}\bigr)\,dt \\ & = \int_{0}^{T}\bigl(u+B^{*}p, u-U_{N}\bigr)\,dt+ \int_{0}^{T}\bigl(U_{N}+B^{*}P(U _{N}), U_{N}-u\bigr)\,dt \\ & = \int_{0}^{T}\bigl(u+B^{*}p, u-U_{N}\bigr)\,dt+ \int_{0}^{T}\bigl(U_{N}+B^{*} \tilde{P}_{N}, U_{N}-u\bigr)\,dt \\ &\quad {} + \int_{0}^{T}\bigl(B^{*}\bigl( \tilde{P}_{N}-p(U_{N})\bigr), u-U_{N}\bigr)\,dt. \end{aligned}$$

Note that $U_{N}\in K^{N}\subset K$. It follows from (2.10) that we have

$$ \int_{0}^{T}\bigl(u+B^{*}p, u-U_{N}\bigr)\,dt\leq 0. $$

Therefore, we can get

3.11 $$\begin{aligned} \begin{aligned}[b] c \Vert u-U_{N} \Vert ^{2}_{L^{2}(0,T;L^{2}(\Omega ))}&\leq \int_{0}^{T}\bigl(U_{N}+B ^{*} \tilde{P}_{N}, U_{N}-u\bigr)\,dt \\ & \quad {} + \int_{0}^{T}\bigl(B^{*}\bigl( \tilde{P}_{N}-p(U _{N})\bigr), u-U_{N}\bigr)\,dt \\ &\equiv E_{1}+E_{2}. \end{aligned} \end{aligned}$$

Firstly, we can easily estimate the first term $E_{1}$ of (3.11) as follows:

3.12 $$\begin{aligned} E_{1}&= \int_{0}^{T}\bigl(U_{N}+B^{*} \tilde{P}_{N}, U_{N}-u\bigr)\,dt=\sum _{i=1}^{M} \int_{t_{i-1}}^{t_{i}}\bigl(U_{N}+B^{*} \tilde{P}_{N}, U_{N}-u\bigr)\,dt \\ &\leq C(\delta )\sum_{i=1}^{M} \int_{t_{i-1}}^{t_{i}} \bigl\Vert U_{N}+B ^{*}\tilde{P}_{N} \bigr\Vert _{L^{2}(\Omega )}^{2}\,dt +\delta \sum_{i=1} ^{M} \int_{t_{i-1}}^{t_{i}} \Vert U_{N}-u \Vert _{L^{2}(\Omega )}^{2}\,dt \\ &\leq C( \delta )\eta_{1}^{2}+\delta \Vert u-U_{N} \Vert ^{2}_{L^{2}(0,T;L^{2}(\Omega ))}. \end{aligned}$$

For the second term $E_{2}$, we can also easily obtain

3.13 $$\begin{aligned} \begin{aligned}[b] E_{2}&= \int_{0}^{T}\bigl(B^{*}\bigl( \tilde{P}_{N}-p(U_{N})\bigr), u-U_{N}\bigr)\,dt \\ &\leq C(\delta ) \int_{0}^{T} \bigl\Vert B^{*}( \tilde{P}_{N}-p(U_{N}) \bigr\Vert ^{2}_{L ^{2}(\Omega )}\,dt+ \delta \int_{0}^{T} \Vert U_{N}-u \Vert _{L^{2}(\Omega )}^{2}\,dt \\ &\leq C \bigl\Vert \tilde{P}_{N}-p(U_{N}) \bigr\Vert ^{2}_{L^{2}(0,T;L^{2}(\Omega ))}+ \delta \Vert u-U_{N} \Vert ^{2}_{L^{2}(0,T;L^{2}(\Omega ))}. \end{aligned} \end{aligned}$$

Finally, for any sufficiently small positive number *δ*, and from (3.11)–(3.13), we have

$$ \Vert u-U_{N} \Vert ^{2}_{L^{2}(0,T;L^{2}(\Omega ))}\leq C \eta_{1}^{2}+C \bigl\Vert p(U _{N})- \tilde{P}_{N} \bigr\Vert ^{2}_{L^{2}(0,T;L^{2}(\Omega ))}. $$

This proves (3.10). □

### Theorem 3.2

*Let* $(Y_{N},P_{N},U_{N})$ *be the solutions of* (2.25)*–*(2.29), *let* $(y(U_{N}),p(U_{N}))$ *be defined by* (3.4)*–*(3.7). *Then we have*

3.14 $$ \bigl\Vert Y_{N}-y(U_{N}) \bigr\Vert ^{2}_{L^{2}(0,T;H^{1}(\Omega ))}+ \bigl\Vert P_{N}-p(U_{N}) \bigr\Vert ^{2}_{L^{2}(0,T;H^{1}(\Omega ))}\leq C\sum_{i=2}^{10} \eta_{i} ^{2}, $$

*where*

$$\begin{aligned}& \eta_{2}^{2} = \int_{0}^{T}N^{-2} \int_{\Omega }\bigl(\hat{Y}_{N}-\hat{y} _{d}+ \operatorname{div}\bigl(A^{*}\nabla \tilde{P}_{N}\bigr)-\phi '(\tilde{Y}_{N}) \tilde{P}_{N}+P_{Nt} \bigr)^{2}\,dx\,dt, \\& \eta_{3}^{2} = \int_{0}^{T} \int_{\Omega } \bigl\vert A^{*}\nabla (\tilde{P} _{N}-P_{N}) \bigr\vert ^{2}\,dx\,dt, \\& \eta_{4}^{2} = \Vert y_{d}- \hat{y}_{d} \Vert _{L^{2}(0,T;L^{2}(\Omega ))} ^{2}, \\& \eta_{5}^{2} = \Vert Y_{N}- \tilde{Y}_{N} \Vert _{L^{2}(0,T;L^{2}(\Omega ))} ^{2}, \\& \eta_{6}^{2} = \Vert Y_{N}- \hat{Y}_{N} \Vert _{L^{2}(0,T;L^{2}(\Omega ))} ^{2}, \\& \eta_{7}^{2} = \int_{0}^{T}N^{-2} \int_{\Omega }\bigl(\hat{f}+BU_{N}+ \operatorname{div}(A\nabla \hat{Y}_{N})-\phi (\hat{Y}_{N})-Y_{Nt} \bigr)^{2}\,dx\,dt, \\& \eta_{8}^{2} = \int_{0}^{T} \int_{\Omega } \bigl\vert A\nabla (\hat{Y}_{N}-Y _{N}) \bigr\vert ^{2}\,dx\,dt, \\& \eta_{9}^{2} = \Vert f-\hat{f} \Vert _{L^{2}(0,T;L^{2}(\Omega ))}^{2}, \\& \eta_{10}^{2} = \bigl\Vert Y_{N}(x,0)-y_{0}(x) \bigr\Vert _{L^{2}(\Omega )}^{2}. \end{aligned}$$

### Proof

*Part I*. Let $e^{p}=p(U_{N})-P_{N}$ and $e_{I}^{p}=P^{0}_{1,N}e ^{p}\in V^{N}$, where $P^{0}_{1,N}$ is the orthogonal projection operator defined as in Lemma 2.2. Note that $(p(U_{N})-P _{N})(x,T)=0$, hence

3.15 $$\begin{aligned} & \int_{0}^{T}-\bigl(p_{t}(U_{N})-P_{Nt},e^{p} \bigr)\,dt \\ &\quad =- \int_{0}^{T} \frac{1}{2}\frac{d}{d t} \bigl\Vert p(U_{N})-P_{N} \bigr\Vert _{L^{2}(\Omega )}^{2}\,dt \\ &\quad=- \frac{1}{2} \bigl( \bigl\Vert \bigl(p(U_{N})-P_{N} \bigr) (x,T) \bigr\Vert _{L^{2}(\Omega )}^{2}- \bigl\Vert \bigl(p(U _{N})-P_{N}\bigr) (x,0) \bigr\Vert _{L^{2}(\Omega )}^{2} \bigr) \\ &\quad=\frac{1}{2} \bigl\Vert \bigl(p(U _{N})-P_{N} \bigr) (x,0) \bigr\Vert _{L^{2}(\Omega )}^{2}\geq 0. \end{aligned}$$

By using Eqs. (2.27), (3.6), and (3.15), we have

3.16 $$\begin{aligned} \begin{aligned}[b] & c \bigl\Vert e^{p} \bigr\Vert _{L^{2}(0,T;H^{1}(\Omega ))}^{2} \\ &\quad\leq \int_{0}^{T}a\bigl(e ^{p},p(U_{N})-P_{N} \bigr)\,dt+ \int_{0}^{T}\bigl(\phi ' \bigl(y(U_{N})\bigr) \bigl(p(U_{N})-P_{N}\bigr),e ^{p}\bigr)\,dt \\ &\quad\leq \int_{0}^{T}\bigl(\nabla e^{p},A^{*} \nabla \bigl(p(U_{N})-P_{N}\bigr)\bigr)\,dt- \int_{0}^{T}\bigl(p_{t}(U_{N})-P_{Nt},e^{p} \bigr)\,dt \\ &\quad\quad {} + \int_{0}^{T}\bigl(\phi '\bigl(y(U _{N})\bigr) \bigl(p(U_{N})-\tilde{P}_{N} \bigr),e^{p}\bigr)\,dt \\ &\quad\quad {} + \int_{0}^{T}\bigl(\phi '\bigl(y(U _{N})\bigr) (\tilde{P}_{N}-P_{N}),e^{p} \bigr)\,dt \\ &\quad= \int_{0}^{T}\bigl(\nabla e^{p},A ^{*}\nabla \bigl(p(U_{N})-\tilde{P}_{N}\bigr) \bigr)\,dt- \int_{0}^{T}\bigl(p_{t}(U_{N})-P _{Nt},e^{p}\bigr)\,dt \\ &\quad\quad {} + \int_{0}^{T}\bigl(\nabla e^{p},A^{*} \nabla (\tilde{P} _{N}-P_{N})\bigr)\,dt \\ &\quad\quad {} + \int_{0}^{T}\bigl(\phi ' \bigl(y(U_{N})\bigr)p(U_{N})-\phi '( \tilde{Y}_{N})\tilde{P}_{N},e^{p}\bigr)\,dt \\ &\quad\quad {} + \int_{0}^{T}\bigl(\tilde{\phi }''( \tilde{Y}_{N}) \bigl(\tilde{Y}_{N}-y(U_{N}) \bigr)\tilde{P}_{N},e^{p}\bigr)\,dt \\ &\quad\quad {} + \int _{0}^{T}\bigl(\phi ' \bigl(y(U_{N})\bigr) (\tilde{P}_{N}-P_{N}),e^{p} \bigr)\,dt. \end{aligned} \end{aligned}$$

From (3.16), we can get

3.17 $$\begin{aligned} \begin{aligned}[b] & c \bigl\Vert e^{p} \bigr\Vert _{L^{2}(0,T;H^{1}(\Omega ))}^{2} \\ &\quad\leq \int_{0}^{T}\bigl( \nabla \bigl(e^{p}-e_{I}^{p} \bigr),A^{*}\nabla \bigl(p(U_{N})-\tilde{P}_{N} \bigr)\bigr)\,dt- \int _{0}^{T}\bigl(p_{t}(U_{N})-P_{Nt},e^{p}-e_{I}^{p} \bigr)\,dt \\ &\quad\quad {} + \int_{0}^{T}\bigl( \phi ' \bigl(y(U_{N})\bigr)p(U_{N})-\phi '( \tilde{Y}_{N})\tilde{P}_{N},e^{p}-e _{I}^{p}\bigr)\,dt \\ &\quad \quad {} + \int_{0}^{T}\bigl(\nabla e_{I}^{p},A^{*} \nabla \bigl(p(U_{N})- \tilde{P}_{N}\bigr)\bigr)\,dt- \int_{0}^{T}\bigl(p_{t}(U_{N})-P_{Nt},e_{I}^{p} \bigr)\,dt \\ &\quad \quad {} + \int_{0}^{T}\bigl(\phi ' \bigl(y(U_{N})\bigr)p(U_{N})-\phi '( \tilde{Y}_{N})\tilde{P} _{N},e_{I}^{p} \bigr)\,dt \\ &\quad \quad {} + \int_{0}^{T}\bigl(\tilde{\phi }''( \tilde{Y}_{N}) \bigl( \tilde{Y}_{N}-y(U_{N}) \bigr)\tilde{P}_{N},e^{p}\bigr)\,dt \\ &\quad\quad {} + \int_{0}^{T}\bigl(\phi '\bigl(y(U _{N})\bigr) (\tilde{P}_{N}-P_{N}),e^{p} \bigr)\,dt+ \int_{0}^{T}\bigl(\nabla e^{p},A^{*} \nabla (\tilde{P}_{N}-P_{N})\bigr)\,dt. \end{aligned} \end{aligned}$$

Thanks to $e^{p}-e_{I}^{p}\in V=H_{0}^{1}(\Omega )$, from Eqs. (2.27), (3.6), and (3.17), we can obtain

3.18 $$\begin{aligned} \begin{aligned}[b] & c \bigl\Vert e^{p} \bigr\Vert _{L^{2}(0,T;H^{1}(\Omega ))}^{2} \\ &\quad= \int_{0}^{T}\bigl(y(U_{N})-y _{d}+\operatorname{div}\bigl(A^{*}\nabla \tilde{P}_{N} \bigr)-\phi '(\tilde{Y}_{N}) \tilde{P}_{N}+P_{Nt},e^{p}-e_{I}^{p} \bigr)\,dt \\ &\quad\quad {} + \int_{0}^{T}\bigl(\tilde{\phi }''( \tilde{Y}_{N}) \bigl(\tilde{Y}_{N}-y(U_{N}) \bigr)\tilde{P}_{N},p(U_{N})-P_{N}\bigr)\,dt \\ &\quad\quad {} + \int_{0}^{T}\bigl(y(U_{N})- \hat{Y}_{N},e_{I}^{p}\bigr)\,dt+ \int_{0}^{T}\bigl( \hat{y}_{d}-y_{d},e_{I}^{p} \bigr)\,dt \\ &\quad\quad {} + \int_{0}^{T}\bigl(\phi ' \bigl(y(U_{N})\bigr) ( \tilde{P}_{N}-P_{N}),p(U_{N})-P_{N} \bigr)\,dt \\ &\quad\quad {}+ \int_{0}^{T}\bigl(\nabla e^{p},A ^{*}\nabla (\tilde{P}_{N}-P_{N})\bigr)\,dt. \end{aligned} \end{aligned}$$

Then we have

3.19 $$\begin{aligned} \begin{aligned}[b] & c \bigl\Vert e^{p} \bigr\Vert _{L^{2}(0,T;H^{1}(\Omega ))}^{2} \\ &\quad\leq \int_{0}^{T}\bigl( \hat{Y}_{N}- \hat{y}_{d}+\operatorname{div}\bigl(A^{*}\nabla \tilde{P}_{N}\bigr)-\phi '( \tilde{Y}_{N}) \tilde{P}_{N}+P_{Nt},e^{p}-e_{I}^{p} \bigr)\,dt \\ &\quad\quad {} + \int_{0} ^{T}\bigl(\phi ' \bigl(y(U_{N})\bigr) (\tilde{P}_{N}-P_{N}),p(U_{N})-P_{N} \bigr)\,dt+ \int_{0} ^{T}\bigl(\nabla e^{p},A^{*} \nabla (\tilde{P}_{N}-P_{N})\bigr)\,dt \\ &\quad\quad {} + \int_{0} ^{T}\bigl(\hat{y}_{d}-y_{d},e^{p} \bigr)\,dt+ \int_{0}^{T}\bigl(\tilde{\phi }''( \tilde{Y}_{N}) \bigl(\tilde{Y}_{N}-y(U_{N}) \bigr)\tilde{P}_{N},P(U_{N})-P_{N}\bigr)\,dt \\ &\quad\quad {} + \int_{0}^{T}\bigl(y(U_{N})- \hat{Y}_{N},e^{p}\bigr)\,dt \\ &\quad \equiv \sum_{i=1}^{6}I_{i}. \end{aligned} \end{aligned}$$

By using Lemma 2.2, we can estimate the first term $I_{1}$ as follows:

3.20 $$\begin{aligned} \begin{aligned}[b] I_{1}&= \int_{0}^{T}\bigl(\hat{Y}_{N}- \hat{y}_{d}+\operatorname{div}\bigl(A^{*}\nabla \tilde{P}_{N}\bigr)-\phi '(\tilde{Y}_{N}) \tilde{P}_{N}+P_{Nt},e^{p}-e_{I} ^{p}\bigr)\,dt \\ &\leq C(\delta ) \int_{0}^{T}N^{-2} \int_{\Omega }\bigl(\hat{Y} _{N}-\hat{y}_{d}+ \operatorname{div}\bigl(A^{*}\nabla \tilde{P}_{N}\bigr)-\phi '( \tilde{Y}_{N})\tilde{P}_{N}+P_{Nt} \bigr)^{2}\,dx\,dt \\ &\quad {} +\delta \int_{0}^{T} \bigl\Vert e ^{p} \bigr\Vert _{H^{1}(\Omega )}^{2}\,dt \\ &\leq C(\delta )\eta_{2}^{2}+\delta \bigl\Vert p(U_{N})-P_{N} \bigr\Vert _{L^{2}(0,T;H^{1}(\Omega ))}^{2}, \end{aligned} \end{aligned}$$

where *δ* is an arbitrary positive number, $C(\delta )$ is a constant dependent on *δ*. Note that $\phi '(\cdot )\geq 0$, we can obtain

3.21 $$\begin{aligned} I_{2}&= \int_{0}^{T}\bigl(\phi ' \bigl(y(U_{N})\bigr) (\tilde{P}_{N}-P_{N}),p(U_{N})-P _{N}\bigr)\,dt \\ &\leq C(\delta ) \Vert \tilde{P}_{N}-P_{N} \Vert _{L^{2}(0,T;L^{2}( \Omega ))}^{2}+\delta \bigl\Vert p(U_{N})-P_{N} \bigr\Vert _{L^{2}(0,T;L^{2}(\Omega ))} ^{2} \\ &\leq C(\delta ) \int_{0}^{T} \int_{\Omega } \bigl\vert A^{*}\nabla ( \tilde{P}_{N}-P_{N}) \bigr\vert ^{2}\,dx\,dt+\delta \bigl\Vert p(U_{N})-P_{N} \bigr\Vert _{L^{2}(0,T;H ^{1}(\Omega ))}^{2} \\ &\leq C(\delta )\eta_{3}^{2}+\delta \bigl\Vert p(U_{N})-P _{N} \bigr\Vert _{L^{2}(0,T;H^{1}(\Omega ))}^{2}. \end{aligned}$$

For the third term $I_{3}$, we can derive

3.22 $$\begin{aligned} \begin{aligned}[b] I_{3}&= \int_{0}^{T}\bigl(\nabla e^{p},A^{*} \nabla (\tilde{P}_{N}-P_{N})\bigr)\,dt \\ &\leq C(\delta ) \int_{0}^{T} \int_{\Omega } \bigl\vert A^{*}\nabla (\tilde{P} _{N}-P_{N}) \bigr\vert ^{2}\,dx\,dt+\delta \int_{0}^{T} \int_{\Omega } \bigl\vert \nabla e^{p} \bigr\vert ^{2}\,dx\,dt \\ &\leq C(\delta )\eta_{3}^{2}+\delta \bigl\Vert p(U_{N})-P_{N} \bigr\Vert _{L^{2}(0,T;H ^{1}(\Omega ))}^{2}. \end{aligned} \end{aligned}$$

Similarly, for $I_{4}$ we have the following estimate:

3.23 $$\begin{aligned} \begin{aligned}[b] I_{4}&= \int_{0}^{T}\bigl(\hat{y}_{d}-y_{d},e^{p} \bigr)\,dt \\ &\leq C(\delta ) \Vert y _{d}-\hat{y}_{d} \Vert _{L^{2}(0,T;L^{2}(\Omega ))}^{2}+\delta \bigl\Vert p(U_{N})-P _{N} \bigr\Vert _{L^{2}(0,T;L^{2}(\Omega ))}^{2} \\ &\leq C(\delta )\eta^{2}_{4}+ \delta \bigl\Vert p(U_{N})-P_{N} \bigr\Vert _{L^{2}(0,T;H^{1}(\Omega ))}^{2}. \end{aligned} \end{aligned}$$

Due to $\phi (\cdot )\in W^{2,\infty }(-R,-R)$, we can get

3.24 $$\begin{aligned} \begin{aligned}[b] I_{5}&= \int_{0}^{T}\bigl(\tilde{\phi }''( \tilde{Y}_{N}) \bigl(\tilde{Y}_{N}-y(U _{N}) \bigr)\tilde{P}_{N},p(U_{N})-P_{N}\bigr)\,dt \\ &\leq C(\delta ) \int_{0}^{T} \int_{\Omega } \bigl\vert y(U_{N})- \tilde{Y}_{N} \bigr\vert ^{2}\,dx\,dt+\delta \bigl\Vert p(U_{N})-P _{N} \bigr\Vert _{L^{2}(0,T;L^{2}(\Omega ))}^{2} \\ &\leq C(\delta ) \bigl\Vert y(U_{N})-Y _{N} \bigr\Vert _{L^{2}(0,T;L^{2}(\Omega ))}^{2}+C(\delta ) \Vert Y_{N}- \tilde{Y} _{N} \Vert _{L^{2}(0,T;L^{2}(\Omega ))}^{2} \\ & \quad {} +\delta \bigl\Vert p(U_{N})-P_{N} \bigr\Vert _{L^{2}(0,T;H^{1}(\Omega ))}^{2} \\ &\leq C(\delta )\eta_{5}^{2}+C( \delta ) \bigl\Vert y(U_{N})-Y_{N} \bigr\Vert _{L^{2}(0,T;H^{1}(\Omega ))}^{2}+ \delta \bigl\Vert p(U _{N})-P_{N} \bigr\Vert _{L^{2}(0,T;H^{1}(\Omega ))}^{2}. \end{aligned} \end{aligned}$$

Then we can estimate the last term $I_{6}$ of (3.19) as follows:

3.25 $$\begin{aligned} \begin{aligned}[b] I_{6}&= \int_{0}^{T}\bigl(y(U_{N})- \hat{Y}_{N},e^{p}\bigr)\,dt \\ &\leq C(\delta ) \int_{0}^{T} \int_{\Omega } \bigl\vert y(U_{N})- \hat{Y}_{N} \bigr\vert ^{2}\,dx\,dt+\delta \bigl\Vert p(U _{N})-P_{N} \bigr\Vert _{L^{2}(0,T;L^{2}(\Omega ))}^{2} \\ &\leq C(\delta ) \bigl\Vert y(U _{N})-Y_{N} \bigr\Vert _{L^{2}(0,T;L^{2}(\Omega ))}^{2}+C(\delta ) \Vert Y_{N}- \hat{Y}_{N} \Vert _{L^{2}(0,T;L^{2}(\Omega ))}^{2} \\ &\quad {} +\delta \bigl\Vert p(U_{N})-P _{N} \bigr\Vert _{L^{2}(0,T;H^{1}(\Omega ))}^{2} \\ &\leq C(\delta )\eta_{6}^{2}+C( \delta ) \bigl\Vert y(U_{N})-Y_{N} \bigr\Vert _{L^{2}(0,T;H^{1}(\Omega ))}^{2}+ \delta \bigl\Vert p(U _{N})-P_{N} \bigr\Vert _{L^{2}(0,T;H^{1}(\Omega ))}^{2} . \end{aligned} \end{aligned}$$

Therefore, let *δ* be small enough, from (3.16)–(3.25), we obtain

3.26 $$ \bigl\Vert p(U_{N})-P_{N} \bigr\Vert _{L^{2}(0,T;H^{1}(\Omega ))}^{2}\leq C(\delta ) \sum_{i=2}^{6} \eta_{i}^{2}+C(\delta ) \bigl\Vert y(U_{N})-Y_{N} \bigr\Vert _{L^{2}(0,T;H ^{1}(\Omega ))}^{2}. $$

*Part II*. Let $e^{y}=y(U_{N})-Y_{N}$, $e_{I}^{y}=P^{0}_{1,N} e ^{y}\in V^{N}$, where $P^{0}_{1,N}$ is the orthogonal projection operator defined as in Lemma 2.2. Note that

$$\begin{aligned} \begin{aligned}[b] \int_{0}^{T}\bigl(y_{t}(U_{N})-Y_{Nt},e^{y} \bigr)\,dt&= \int_{\Omega } \int_{0}^{T}e ^{y}\bigl(y_{t}(U_{N})-Y_{Nt} \bigr)\,dt\,dx \\ &= \int_{\Omega } \int_{0}^{T}e^{y}\frac{d(y(U_{N})-Y_{N})}{dt}\,dt\,dx \\ &= \int_{\Omega } \int_{0}^{T}e^{y}\,d\bigl(y(U_{N})-Y_{N} \bigr)\,dx \\ &= \int_{\Omega }\bigl(\bigl(y(U_{N})-Y_{N}\bigr) (x,T)\bigr)^{2}\,dx- \int_{\Omega }\bigl(\bigl(y(U _{N})-Y_{N} \bigr) (x,0)\bigr)^{2}\,dx \\ &\quad {} - \int_{0}^{T}\bigl(y_{t}(U_{N})-Y_{Nt},e^{y} \bigr)\,dt, \end{aligned} \end{aligned}$$

then we have

$$\begin{aligned} \begin{aligned} \int_{0}^{T}\bigl(y_{t}(U_{N})-Y_{Nt},e^{y} \bigr)\,dt&=\frac{1}{2} \int_{\Omega }\bigl(\bigl(y(U _{N})-Y_{N} \bigr) (x,T)\bigr)^{2}\,dx\\&\quad{} -\frac{1}{2} \int_{\Omega }\bigl(\bigl(y(U_{N})-Y_{N}\bigr) (x,0)\bigr)^{2}\,dx.\end{aligned} \end{aligned}$$

Thus

3.27 $$\begin{aligned} \int_{0}^{T}\bigl(y_{t}(U_{N})-Y_{Nt},e^{y} \bigr)\,dt+\frac{1}{2} \bigl\Vert y_{0}(x)-Y_{N}(x,0) \bigr\Vert _{L^{2}(\Omega )}^{2}\geq 0. \end{aligned}$$

From (2.30), it is easy to show that

3.28 $$\begin{aligned} \bigl(\phi \bigl(y(U_{N})\bigr)-\phi (Y_{N}),e^{y}\bigr)=\bigl(\tilde{\phi }' \bigl(y(U_{N})\bigr) \bigl(y(U _{N})-Y_{N} \bigr),e^{y}\bigr)\geq 0. \end{aligned}$$

By using (3.27), we can get

3.29 $$\begin{aligned} \begin{aligned}[b] c \bigl\Vert e^{y} \bigr\Vert _{L^{2}(0,T;H^{1}(\Omega ))}^{2}&\leq \int_{0}^{T}a\bigl(y(U_{N})-Y _{N},e^{y}\bigr)\,dt+ \int_{0}^{T}\bigl(\phi \bigl(y(U_{N}) \bigr)-\phi (Y_{N}),e^{y}\bigr)\,dt \\ &\leq \int_{0}^{T}a\bigl(y(U_{N})-Y_{N},e^{y} \bigr)\,dt+ \int_{0}^{T}\bigl(\phi \bigl(y(U _{N}) \bigr)-\phi (\hat{Y}_{N}),e^{y}\bigr)\,dt \\ & \quad {} + \int_{0}^{T}\bigl(y_{t}(U_{N})-Y_{Nt},e ^{y}\bigr)\,dt+ \int_{0}^{T}\bigl(\phi (\hat{Y}_{N})-\phi (Y_{N}),e^{y}\bigr)\,dt \\ & \quad {} + \frac{1}{2} \bigl\Vert y_{0}(x)-Y_{N}(x,0) \bigr\Vert _{L^{2}(\Omega )}^{2} \\ &= \int_{0} ^{T}\bigl(A\nabla \bigl(y(U_{N})- \hat{Y}_{N}\bigr),\nabla e^{y}\bigr)\,dt+ \int_{0}^{T}\bigl( \phi \bigl(y(U_{N}) \bigr)-\phi (\hat{Y}_{N}),e^{y}\bigr)\,dt \\ & \quad {} + \int_{0}^{T}\bigl(A\nabla (\hat{Y}_{N}-Y_{N}), \nabla e^{y}\bigr)\,dt+ \int_{0}^{T}\bigl(y_{t}(U_{N})-Y_{Nt},e ^{y}\bigr)\,dt \\ &\quad {} + \int_{0}^{T}\bigl(\phi (\hat{Y}_{N})-\phi (Y_{N}),e^{y}\bigr)\,dt+ \frac{1}{2} \bigl\Vert y_{0}(x)-Y_{N}(x,0) \bigr\Vert _{L^{2}(\Omega )}^{2}. \end{aligned} \end{aligned}$$

Combining (3.28) and (3.29), note that $e^{y}-e_{I}^{y} \in H_{0}^{1}(\Omega )$, from (2.25) and (3.4), we can obtain

3.30 $$\begin{aligned} c \bigl\Vert e^{y} \bigr\Vert _{L^{2}(0,T;H^{1}(\Omega ))}^{2}&\leq \int_{0}^{T}\bigl(\hat{f}+BU _{N}+ \operatorname{div}(A\nabla \hat{Y}_{N})-\phi (\tilde{Y}_{N})-Y_{Nt},e^{y}-e _{I}^{y}\bigr)\,dt \\ & \quad {} + \int_{0}^{T}\bigl(\tilde{\phi }'( \hat{Y}_{N}) (\hat{Y}_{N}-Y _{N}),e^{y} \bigr)\,dt+ \int_{0}^{T}\bigl(A\nabla (\hat{Y}_{N}-Y_{N}), \nabla e^{y}\bigr)\,dt \\ & \quad {} + \int_{0}^{T}\bigl(f-\hat{f},e^{y} \bigr)\,dt+\frac{1}{2} \bigl\Vert Y_{N}(x,0)-y_{0}(x) \bigr\Vert _{L^{2}(\Omega )}^{2} \\ &\equiv J_{1}+J_{2}+J_{3}+J_{4}+ \frac{1}{2} \eta_{10}^{2}. \end{aligned}$$

From Lemma (2.2), we have

3.31 $$\begin{aligned} \begin{aligned}[b] J_{1}&= \int_{0}^{T}\bigl(\hat{f}+BU_{N}+ \operatorname{div}(A\nabla \hat{Y}_{N})- \phi (\tilde{Y}_{N})-Y_{Nt},e^{y}-e_{I}^{y} \bigr)\,dt \\ &\leq C(\delta ) \int _{0}^{T}N^{-2} \int_{\Omega }\bigl(\hat{f}+BU_{N}+\operatorname{div}(A\nabla \hat{Y} _{N})-\phi (\tilde{Y}_{N})-Y_{Nt} \bigr)^{2}\,dx\,dt \\ & \quad {} +\delta \int_{0}^{T} \bigl\Vert e ^{y} \bigr\Vert _{H^{1}(\Omega )}^{2}\,dt \\ &\leq C(\delta )\eta_{7}^{2}+\delta \bigl\Vert y(U_{N})-Y_{N} \bigr\Vert _{L^{2}(0,T;H^{1}(\Omega ))}^{2}. \end{aligned} \end{aligned}$$

For $J_{2}$, by using the assumption of *ϕ*, we can get

3.32 $$\begin{aligned} \begin{aligned}[b] J_{2}&= \int_{0}^{T}\bigl(\tilde{\phi }'( \hat{Y}_{N}) (\hat{Y}_{N}-Y_{N}),e ^{y}\bigr)\,dt \\ &\leq C(\delta ) \Vert Y_{N}-\hat{Y}_{N} \Vert _{L^{2}(0,T;L^{2}( \Omega ))}^{2}+\delta \bigl\Vert e^{y} \bigr\Vert _{L^{2}(0,T;L^{2}(\Omega ))}^{2} \\ &\leq C(\delta )\eta_{6}^{2}+\delta \bigl\Vert e^{y} \bigr\Vert _{L^{2}(0,T;H^{1}(\Omega ))}^{2}. \end{aligned} \end{aligned}$$

For $J_{3}$, it is clear that

3.33 $$\begin{aligned} \begin{aligned}[b] J_{3}&= \int_{0}^{T}\bigl(A\nabla (\hat{Y}_{N}-Y_{N}), \nabla e^{y}\bigr)\,dt \\ &\leq C(\delta ) \int_{0}^{T} \int_{\Omega } \bigl\vert A\nabla (\hat{Y}_{N}-Y_{N}) \bigr\vert ^{2}\,dx\,dt+ \delta \int_{0}^{T} \int_{\Omega } \bigl\vert A\nabla e^{y} \bigr\vert ^{2}\,dx\,dt \\ &\leq C( \delta )\eta_{8}^{2}+\delta \bigl\Vert y(U_{N})-Y_{N} \bigr\Vert _{L^{2}(0,T;H^{1}( \Omega ))}^{2}. \end{aligned} \end{aligned}$$

For $J_{4}$, we can obtain

3.34 $$\begin{aligned} \begin{aligned}[b] J_{4}&= \int_{0}^{T}\bigl(f-\hat{f},e^{y}\bigr)\,dt \\ &\leq C(\delta ) \Vert f-\hat{f} \Vert _{L^{2}(0,T;L^{2}(\Omega ))}^{2}+\delta \bigl\Vert y(U_{N})-Y_{N} \bigr\Vert _{L^{2}(0,T;L ^{2}(\Omega ))}^{2} \\ &\leq C(\delta )\eta_{9}^{2}+\delta \bigl\Vert y(U_{N})-Y _{N} \bigr\Vert _{L^{2}(0,T;H^{1}(\Omega ))}^{2}. \end{aligned} \end{aligned}$$

By simplifying both sides of (3.30), we get

3.35 $$ \bigl\Vert y(U_{N})-Y_{N} \bigr\Vert _{L^{2}(0,T;H^{1}(\Omega ))}^{2}\leq C(\delta ) \sum_{i=6}^{10} \eta_{i}^{2}. $$

Then we derive (3.14) follows from (3.26) and (3.35). □

### Theorem 3.3

*Let* $(y,p,u)$ *and* $(Y_{N},P_{N},U_{N})$ *be the solutions of* (2.8)*–*(2.10) *and* (2.25)*–*(2.29), *respectively*. *Assume that all the conditions in Theorem *3.1 *and Theorem *3.2 *are valid*. *Then*

3.36 $$ \Vert Y_{N}-y \Vert _{L^{2}(0,T;H^{1}(\Omega ))}^{2}+ \Vert P_{N}-p \Vert _{L^{2}(0,T;H ^{1}(\Omega ))}^{2}+ \Vert U_{N}-u \Vert _{L^{2}(0,T;L^{2}(\Omega ))}^{2}\leq C \sum _{i=1}^{10}\eta_{i}^{2}, $$

*where* $\eta_{i}^{2}$, $i=1,\ldots,10$, *are defined in Theorem *3.1 *and Theorem *3.2.

### Proof

Combining Theorem 3.1 and Theorem 3.2, we have

3.37 $$\begin{aligned} \begin{aligned}[b] \Vert u-U_{N} \Vert _{L^{2}(0,T;L^{2}(\Omega ))}^{2}&\leq C\eta_{1}^{2}+C \bigl\Vert \tilde{P}_{N}-p(U_{N}) \bigr\Vert _{L^{2}(0,T;L^{2}(\Omega ))}^{2} \\ &\leq C \eta_{1}^{2}+C \Vert \tilde{P}_{N}-P_{N} \Vert _{L^{2}(0,T;L^{2}(\Omega ))}^{2}+C \bigl\Vert P_{N}-p(U_{N}) \bigr\Vert _{L^{2}(0,T;L^{2}(\Omega ))}^{2} \\ &\leq C\sum_{i=1}^{10} \eta_{i}^{2}+C \Vert \tilde{P}_{N}-P_{N} \Vert _{L^{2}(0,T;L ^{2}(\Omega ))}^{2}. \end{aligned} \end{aligned}$$

Thanks to *A* being positive definite, according to the Poincaré inequality, we can obtain

3.38 $$ \Vert \tilde{P}_{N}-P_{N} \Vert _{L^{2}(0,T;L^{2}(\Omega ))}^{2}\leq C \int_{0} ^{T} \int_{\Omega } \bigl\vert A^{*}\nabla ( \tilde{P}_{N}-P_{N}) \bigr\vert ^{2}\,dx\,dt=C\eta _{3}^{2}. $$

Plugging (3.38) into (3.37) yields

3.39 $$ \Vert u-U_{N} \Vert _{L^{2}(0,T;L^{2}(\Omega ))}^{2} \leq C\sum_{i=1}^{10}\eta _{i}^{2}. $$

Note that

3.40 $$\begin{aligned}& \Vert Y_{N}-y \Vert _{L^{2}(0,T;H^{1}(\Omega ))}^{2} \leq \bigl\Vert Y_{N}-y(U_{N}) \bigr\Vert _{L^{2}(0,T;H^{1}(\Omega ))}^{2} + \bigl\Vert y(U_{N})-y \bigr\Vert _{L^{2}(0,T;H^{1}( \Omega ))}^{2}, \end{aligned}$$

3.41 $$\begin{aligned}& \Vert P_{N}-p \Vert _{L^{2}(0,T;H^{1}(\Omega ))}^{2} \leq \bigl\Vert P_{N}-p(U_{N}) \bigr\Vert _{L^{2}(0,T;H^{1}(\Omega ))}^{2} + \bigl\Vert p(U_{N})-p \bigr\Vert _{L^{2}(0,T;H^{1}( \Omega ))}^{2}, \end{aligned}$$

and

3.42 $$\begin{aligned}& \bigl\Vert y(U_{N})-y \bigr\Vert _{L^{2}(0,T;H^{1}(\Omega ))}^{2}\leq C \Vert u-U_{N} \Vert _{L ^{2}(0,T;L^{2}(\Omega ))}^{2}, \end{aligned}$$

3.43 $$\begin{aligned}& \bigl\Vert p(U_{N})-p \bigr\Vert _{L^{2}(0,T;H^{1}(\Omega ))}^{2}\leq \bigl\Vert y(U_{N})-y \bigr\Vert _{L ^{2}(0,T;L^{2}(\Omega ))}^{2}\leq C \Vert u-U_{N} \Vert _{L^{2}(0,T;L^{2}( \Omega ))}^{2}. \end{aligned}$$

From (3.39), (3.40), (3.42), and Theorem 3.2, we can derive

3.44 $$\begin{aligned} \begin{aligned}[b] \Vert Y_{N}-y \Vert _{L^{2}(0,T;H^{1}(\Omega ))}^{2}&\leq \bigl\Vert Y_{N}-y(U_{N}) \bigr\Vert _{L^{2}(0,T;H^{1}(\Omega ))}^{2} +C \Vert u-U_{N} \Vert _{L^{2}(0,T;L^{2}( \Omega ))}^{2} \\ &\leq C\sum_{i=1}^{10}\eta_{i}^{2}. \end{aligned} \end{aligned}$$

Similarly, from (3.39), (3.41), (3.43), and Theorem 3.2, we also can derive

3.45 $$\begin{aligned} \Vert P_{N}-p \Vert _{L^{2}(0,T;H^{1}(\Omega ))}^{2} \leq C\sum_{i=1}^{10} \eta _{i}^{2}. \end{aligned}$$

Therefore, (3.36) follows from (3.39) and (3.44)–(3.45). □

## $L^{2}(L^{2})-L^{2}(L^{2})$ a posteriori error estimates

In this section, we shall carry out $L^{2}(L ^{2})-L^{2}(L^{2})$ a posteriori error estimates for the spectral approximation of the optimal control problem governed by nonlinear parabolic equations. In order to estimate the error $\Vert \tilde{P}_{N}-p(U _{N}) \Vert ^{2}_{L^{2}(0,T;L^{2}(\Omega ))}$, we shall use two auxiliary equations.

Now, we set the following dual auxiliary equations:

4.1 $$ \textstyle\begin{cases} -\frac{\partial \zeta }{\partial t}-\operatorname{div}(A^{*}\nabla {\zeta })+ \Phi \zeta =F, & x\in \Omega ,t\in (0,T]; \\ \zeta | _{\partial \Omega }=0, & t\in [0,T]; \\ \zeta (x,0)=0, & x\in \Omega , \end{cases} $$

and

4.2 $$ \textstyle\begin{cases} \frac{\partial \xi }{\partial t}-\operatorname{div}(A\nabla {\xi })+\phi '(y(U _{N}))\xi =F, & x\in \Omega , t\in (0,T]; \\ \xi | _{\partial \Omega }=0, & t\in [0,T]; \\ \xi (x,T)=0, & x\in \Omega , \end{cases} $$

where

$$ \Phi = \textstyle\begin{cases} \frac{\phi (y(U_{N}))-\phi (Y_{N})}{y(U_{N})-Y_{N}}, & y(U_{N})\neq Y _{N} , \\ \phi '(Y_{N}), & y(U_{N})= Y_{N} . \end{cases} $$

The following well-known stability results are presented in [10].

### Lemma 4.1

*Assume that* Ω *is a convex domain*. *Let* *ζ* *and* *ξ* *be the solutions of* (4.1) *and* (4.2), *respectively*. *Then*, *for* $v=\zeta $ *or* $v=\xi $,

$$\begin{aligned}& \Vert v \Vert _{L^{\infty }(0,T;L^{2}(\Omega ))}\leq C \Vert F \Vert _{L^{2}(0,T;L^{2}( \Omega ))}, \\& \Vert \nabla v \Vert _{L^{2}(0,T;L^{2}(\Omega ))}\leq C \Vert F \Vert _{L^{2}(0,T;L ^{2}(\Omega ))}, \\& \bigl\Vert D^{2} v \bigr\Vert _{L^{2}(0,T;L^{2}(\Omega ))}\leq C \Vert F \Vert _{L^{2}(0,T;L ^{2}(\Omega ))}, \\& \biggl\Vert \frac{\partial v}{\partial t} \biggr\Vert _{L^{2}(0,T;L^{2}( \Omega ))}\leq C \Vert F \Vert _{L^{2}(0,T;L^{2}(\Omega ))}, \end{aligned}$$

*where* $D^{2} v=\partial^{2} v /{\partial x_{i}\partial x_{j}}$, $1 \leq i,j\leq n$.

### Theorem 4.1

*Let* $(Y_{N},P_{N},U_{N})$ *be the solutions of* (2.25)*–*(2.29), *let* $(y(U_{N}),p(U_{N}))$ *be defined by* (3.4)*–*(3.7). *Then*

4.3 $$ \bigl\Vert Y_{N}-y(U_{N}) \bigr\Vert ^{2}_{L^{2}(0,T;L^{2}(\Omega ))}+ \bigl\Vert P_{N}-p(U_{N}) \bigr\Vert ^{2}_{L^{2}(0,T;L^{2}(\Omega ))}\leq C\sum_{i=2}^{10} \hat{\eta }_{i}^{2}, $$

*where*

$$\begin{aligned}& \hat{\eta }^{2}_{2} = \int_{0}^{T}N^{-4} \int_{\Omega }\bigl(\hat{Y}_{N}- \hat{y}_{d}+ \operatorname{div}\bigl(A^{*}\nabla \tilde{P}_{N}\bigr)-\phi '(\tilde{Y}_{N}) \tilde{P}_{N}+P_{Nt} \bigr)^{2}\,dx\,dt, \\& \hat{\eta }^{2}_{3} = \int_{0}^{T} \int_{\Omega } \bigl\vert A^{*}\nabla ( \tilde{P}_{N}-P_{N}) \bigr\vert ^{2}\,dx\,dt, \\& \hat{\eta }^{2}_{4} = \Vert y_{d}- \hat{y}_{d} \Vert _{L^{2}(0,T;L^{2}(\Omega ))}^{2}, \\& \hat{\eta }^{2}_{5} = \Vert Y_{N}- \tilde{Y}_{N} \Vert _{L^{2}(0,T;L^{2}( \Omega ))}^{2}, \\& \hat{\eta }^{2}_{6} = \Vert Y_{N}- \hat{Y}_{N} \Vert _{L^{2}(0,T;L^{2}(\Omega ))}^{2}, \\& \hat{\eta }^{2}_{7} = \int_{0}^{T}N^{-4} \int_{\Omega }\bigl(\hat{f}+BU _{N}+\operatorname{div}(A\nabla \hat{Y}_{N})-\phi (\hat{Y}_{N})-Y_{Nt} \bigr)^{2}\,dx\,dt, \\& \hat{\eta }^{2}_{8} = \int_{0}^{T} \int_{\Omega } \bigl\vert A\nabla (\hat{Y} _{N}-Y_{N}) \bigr\vert ^{2}\,dx\,dt, \\& \hat{\eta }^{2}_{9} = \Vert f-\hat{f} \Vert ^{2}_{L^{2}(0,T;L^{2}(\Omega ))}, \\& \hat{\eta }^{2}_{10} = \bigl\Vert Y_{N}(x,0)-y_{0}(x) \bigr\Vert ^{2}_{L^{2}(\Omega )}. \end{aligned}$$

### Proof

*Part I*. Let *ξ* be the solution of (4.2) with $F= p(U_{N})-P_{N}$. Let $\xi_{I}=P^{0}_{1,N} \xi \in V^{N}$, where $P^{0}_{1,N}$ is the orthogonal projection operator defined as in Lemma 2.2. Note that $\xi -\xi_{I}\in H_{0}^{1}(\Omega )$, it follows from (2.27) and (3.6) that

4.4 $$\begin{aligned}& \bigl\Vert p(U_{N})-P_{N} \bigr\Vert ^{2}_{L^{2}(0,T;L^{2}(\Omega ))} \\& \quad = \int_{0}^{T}\bigl(p(U _{N})-P_{N},F \bigr)\,dt \\& \quad = \int_{0}^{T} \bigl(-\bigl(p_{t}(U_{N})-P_{Nt}, \xi \bigr)+a\bigl( \xi , p(U_{N})-P_{N}\bigr)+\bigl(\phi '\bigl(y(U_{N})\bigr) \bigl(p(U_{N})-P_{N} \bigr),\xi \bigr) \bigr)\,dt \\& \quad = \int_{0}^{T} \bigl( -\bigl(p_{t}(U_{N})-P_{Nt}, \xi -\xi_{I}\bigr)+a\bigl(\xi - \xi_{I}, p(U_{N})-\tilde{P}_{N}\bigr) \bigr) \,dt \\& \quad \quad {} + \int_{0}^{T}\bigl(\phi '\bigl(y(U _{N})\bigr)p(U_{N})-\phi '( \tilde{Y}_{N})\tilde{P}_{N},\xi -\xi_{I}\bigr)\,dt \\& \quad \quad {} + \int_{0}^{T} \bigl( -\bigl(p_{t}(U_{N})-P_{Nt}, \xi_{I}\bigr)+a\bigl(\xi_{I}, p(U_{N})- \tilde{P}_{N}\bigr) \bigr) \,dt \\& \quad \quad {} + \int_{0}^{T} \bigl( a(\xi ,\tilde{P}_{N}-P _{N})+\bigl(\phi '(\tilde{Y}_{N}) \tilde{P}_{N}-\phi '\bigl(y(U_{N})\bigr) \tilde{P} _{N},\xi \bigr) \bigr) \,dt \\& \quad \quad {} + \int_{0}^{T}\bigl(\phi ' \bigl(y(U_{N})\bigr)p(U_{N})-\phi '( \tilde{Y}_{N})\tilde{P}_{N},\xi_{I}\bigr)\,dt \\& \quad \quad {} + \int_{0}^{T}\bigl(\phi ' \bigl(y(U_{N})\bigr) ( \tilde{P}_{N}-P_{N}),\xi \bigr)\,dt. \end{aligned}$$

From (4.4), we have

4.5 $$\begin{aligned} \begin{aligned}[b] & \bigl\Vert p(U_{N})-P_{N} \bigr\Vert ^{2}_{L^{2}(0,T;L^{2}(\Omega ))} \\ &\quad = \int_{0}^{T}\bigl(P _{Nt}+\operatorname{div} \bigl(A^{*}\nabla \tilde{P}_{N}\bigr)+\hat{Y}_{N}- \hat{y}_{d}- \phi '(\tilde{Y}_{N}) \tilde{P}_{N}, \xi -\xi_{I}\bigr)\,dt \\ & \quad\quad {} + \int_{0}^{T}\bigl(y(U _{N})-Y_{N}, \xi \bigr)\,dt+ \int_{0}^{T} \bigl(\phi ' \bigl(y(U_{N})\bigr) (\tilde{P}_{N}-P _{N}),\xi \bigr)\,dt \\ &\quad\quad {} + \int_{0}^{T}a(\xi ,\tilde{P}_{N}-P_{N})\,dt+ \int_{0} ^{T}(\hat{y}_{d}-y_{d}, \xi )\,dt \\ &\quad\quad {} + \int_{0}^{T}\bigl(\tilde{\phi }''( \tilde{Y}_{N}) \bigl(\tilde{Y}_{N}-y(U_{N}) \bigr)\tilde{P}_{N},\xi \bigr)\,dt+ \int_{0} ^{T}(Y_{N}-\hat{Y}_{N}, \xi )\,dt \\ &\quad \equiv \sum_{i=1}^{7}K_{i}. \end{aligned} \end{aligned}$$

By using Lemma 2.2 and Lemma 4.1, we have

4.6 $$\begin{aligned} \begin{aligned}[b] K_{1}&= \int_{0}^{T}\bigl(P_{Nt}+\operatorname{div} \bigl(A^{*}\nabla \tilde{P}_{N}\bigr)+ \hat{Y}_{N}- \hat{y}_{d}-\phi '(\tilde{Y}_{N}) \tilde{P}_{N}, \xi -\xi _{I}\bigr)\,dt \\ &\leq C(\delta ) \int_{0}^{T}N^{-4} \int_{\Omega }\bigl(\hat{Y} _{N}-\hat{y}_{d}+ \operatorname{div}\bigl(A^{*}\nabla \tilde{P}_{N}\bigr)-\phi '( \tilde{Y}_{N})\tilde{P}_{N}+P_{Nt} \bigr)^{2}\,dx\,dt \\ & \quad {} +\delta \int_{0}^{T} \Vert \xi \Vert _{H^{2}(\Omega )}^{2}\,dt \\ &\leq C(\delta )\hat{\eta }_{2}^{2}+ \delta \bigl\Vert p(U_{N})-P_{N} \bigr\Vert _{L^{2}(0,T;L^{2}(\Omega ))}^{2}. \end{aligned} \end{aligned}$$

For $K_{2}$, it is easy to see that

4.7 $$\begin{aligned} \begin{aligned}[b] K_{2}&= \int_{0}^{T}\bigl(y(U_{N})-Y_{N}, \xi \bigr)\,dt \\ &\leq C(\delta ) \bigl\Vert y(U _{N})-Y_{N} \bigr\Vert _{L^{2}(0,T;L^{2}(\Omega ))}^{2}+\delta \Vert \xi \Vert _{L^{2}(0,T;L ^{2}(\Omega ))}^{2} \\ &\leq C(\delta ) \bigl\Vert y(U_{N})-Y_{N} \bigr\Vert _{L^{2}(0,T;L ^{2}(\Omega ))}^{2}+\delta \bigl\Vert p(U_{N})-P_{N} \bigr\Vert _{L^{2}(0,T;L^{2}(\Omega ))}^{2}. \end{aligned} \end{aligned}$$

By using the assumption of *ϕ*, we have

4.8 $$\begin{aligned} \begin{aligned} [b] K_{3}&= \int_{0}^{T}\bigl(\phi ' \bigl(y(U_{N})\bigr) (\tilde{P}_{N}-P_{N}),\xi \bigr)\,dt \\ &\leq C(\delta ) \Vert \tilde{P}_{N}-P_{N} \Vert _{L^{2}(0,T;L^{2}(\Omega ))} ^{2}+\delta \Vert \xi \Vert _{L^{2}(0,T;L^{2}(\Omega ))}^{2} \\ &\leq C( \delta ) \int_{0}^{T} \int_{\Omega } \bigl\vert A^{*}\nabla ( \tilde{P}_{N}-P_{N}) \bigr\vert ^{2}\,dx\,dt+ \delta \bigl\Vert p(U_{N})-P_{N} \bigr\Vert _{L^{2}(0,T;L^{2}(\Omega ))}^{2} \\ &\leq C( \delta )\hat{\eta }_{3}^{2}+\delta \bigl\Vert p(U_{N})-P_{N} \bigr\Vert _{L^{2}(0,T;L ^{2}(\Omega ))}^{2}. \end{aligned} \end{aligned}$$

For $K_{4}$, we have

4.9 $$\begin{aligned} \begin{aligned} [b] K_{4}&= \int_{0}^{T}a(\xi ,\tilde{P}_{N}-P_{N})\,dt \\ &\leq C(\delta ) \int_{0}^{T} \int_{\Omega } \bigl\vert A^{*}\nabla ( \tilde{P}_{N}-P_{N}) \bigr\vert ^{2}\,dx\,dt+ \delta \Vert \xi \Vert _{L^{2}(0,T;L^{2}(\Omega ))}^{2} \\ &\leq C(\delta ) \hat{\eta }_{3}^{2}+\delta \bigl\Vert p(U_{N})-P_{N} \bigr\Vert _{L^{2}(0,T;L^{2}(\Omega ))}^{2}. \end{aligned} \end{aligned}$$

In the same way as (4.7), we have

4.10 $$\begin{aligned} \begin{aligned} [b] K_{5}&= \int_{0}^{T}(\hat{y}_{d}-y_{d}, \xi )\,dt \\ &\leq C(\delta ) \Vert y _{d}-\hat{y}_{d} \Vert _{L^{2}(0,T;L^{2}(\Omega ))}^{2}+\delta \Vert \xi \Vert _{L^{2}(0,T;L^{2}(\Omega ))}^{2} \\ &\leq C(\delta )\hat{\eta }_{4} ^{2}+\delta \bigl\Vert p(U_{N})-P_{N} \bigr\Vert _{L^{2}(0,T;L^{2}(\Omega ))}^{2}. \end{aligned} \end{aligned}$$

Note that $\phi (\cdot )\in W^{2,\infty }(-R,-R)$, we obtain

4.11 $$\begin{aligned} \begin{aligned}[b] K_{6}&= \int_{0}^{T}\bigl(\tilde{\phi }''( \tilde{Y}_{N}) \bigl(\tilde{Y}_{N}-y(U _{N}) \bigr)\tilde{P}_{N},\xi \bigr)\,dt \\ &\leq C(\delta ) \int_{0}^{T} \int_{ \Omega } \bigl\vert y(U_{N})- \tilde{Y}_{N} \bigr\vert ^{2}\,dx\,dt+\delta \Vert \xi \Vert _{L^{2}(0,T;L ^{2}(\Omega ))}^{2} \\ &\leq C(\delta ) \bigl\Vert y(U_{N})-Y_{N} \bigr\Vert _{L^{2}(0,T;L ^{2}(\Omega ))}^{2}+C(\delta ) \Vert Y_{N}- \tilde{Y}_{N} \Vert _{L^{2}(0,T;L ^{2}(\Omega ))}^{2} \\ &\quad {} +\delta \bigl\Vert p(U_{N})-P_{N} \bigr\Vert _{L^{2}(0,T;L^{2}( \Omega ))}^{2} \\ &\leq C(\delta )\hat{\eta }_{5}^{2}+C(\delta ) \bigl\Vert y(U _{N})-Y_{N} \bigr\Vert _{L^{2}(0,T;L^{2}(\Omega ))}^{2}+ \delta \bigl\Vert p(U_{N})-P_{N} \bigr\Vert _{L^{2}(0,T;L^{2}(\Omega ))}^{2}. \end{aligned} \end{aligned}$$

For the last term $K_{7}$, we have

4.12 $$\begin{aligned} \begin{aligned}[b] K_{7}&= \int_{0}^{T}(Y_{N}-\hat{Y}_{N}, \xi )\,dt \\ &\leq C(\delta ) \int _{0}^{T} \int_{\Omega } \vert Y_{N}-\hat{Y}_{N} \vert ^{2}\,dx\,dt+\delta \Vert \xi \Vert _{L ^{2}(0,T;L^{2}(\Omega ))}^{2} \\ &\leq C(\delta )\hat{\eta }_{6}^{2}+ \delta \bigl\Vert p(U_{N})-P_{N} \bigr\Vert _{L^{2}(0,T;L^{2}(\Omega ))}^{2}. \end{aligned} \end{aligned}$$

Putting (4.6)–(4.12) into (4.5), letting *δ* be small enough, we obtain

4.13 $$ \bigl\Vert p(U_{N})-P_{N} \bigr\Vert _{L^{2}(0,T;L^{2}(\Omega ))}^{2}\leq C(\delta ) \sum_{i=2}^{6} \hat{\eta }_{i}^{2}+C(\delta ) \bigl\Vert y(U_{N})-Y_{N} \bigr\Vert _{L^{2}(0,T;L^{2}(\Omega ))}^{2}. $$

*Part II*. Let *ζ* be the solution of (4.1) with $F=y(U_{N})-Y_{N}$. Let $\zeta_{I}=P^{0}_{1,N} \zeta \in V^{N}$, where $P^{0}_{1,N}$ is the orthogonal projection operator defined as in Lemma 2.2. Note that $\zeta -\zeta_{I}\in H_{0}^{1}(\Omega )$, from (2.25) and (3.4), we have

$$\begin{aligned} \begin{aligned} & \bigl\Vert y(U_{N})-Y_{N} \bigr\Vert ^{2}_{L^{2}(0,T;L^{2}(\Omega ))} \\ &\quad = \int_{0}^{T}\bigl(y(U _{N})-Y_{N},F \bigr)\,dt \\ &\quad = \int_{0}^{T} \bigl(\bigl(y_{t}(U_{N})-Y_{Nt}, \zeta \bigr)+a\bigl(y(U_{N})-Y_{N}, \zeta \bigr) \bigr)\,dt \\ &\quad \quad {} + \int_{0}^{T}\bigl(\phi \bigl(y(U_{N}) \bigr)-\phi (Y_{N}),\zeta \bigr)\,dt+\bigl(\bigl(y(U_{N})-Y _{N}\bigr) (x,0),\zeta (x,0)\bigr) \\ &\quad = \int_{0}^{T}\bigl(\hat{f}+BU_{N}+ \operatorname{div}(A\nabla \hat{Y}_{N})- \phi (\hat{Y}_{N})-Y_{Nt}, \zeta -\zeta_{I}\bigr)\,dt \\ &\quad \quad {} + \int_{0}^{T}(f-\hat{f},\zeta )\,dt+ \int_{0}^{T}a(\hat{Y}_{N}-Y_{N}, \zeta )\,dt \\ &\quad \quad {} + \int_{0}^{T}\bigl(\phi (\hat{Y}_{N})-\phi (Y_{N}),\zeta \bigr)\,dt+\bigl(\bigl(y(U_{N})-Y _{N}\bigr) (x,0),\zeta (x,0)\bigr) \\ &\quad \leq C(\delta ) \int_{0}^{T}N^{-4} \int_{\Omega }\bigl(\hat{f}+BU_{N}+ \operatorname{div}(A\nabla \hat{Y}_{N})-\phi (\hat{Y}_{N})-Y_{Nt} \bigr)^{2}\,dx\,dt \\ &\quad \quad {} +C(\delta ) \int_{0}^{T} \int_{\Omega } \bigl\vert A\nabla (\hat{Y}_{N}-Y_{N}) \bigr\vert ^{2}\,dx\,dt+C( \delta ) \Vert f-\hat{f} \Vert ^{2}_{L^{2}(0,T;L^{2}(\Omega ))} \\ &\quad \quad {} +C(\delta ) \Vert Y_{N}-\hat{Y}_{N} \Vert ^{2}_{L^{2}(0,T;L^{2}(\Omega ))}+C( \delta ) \bigl\Vert Y_{N}(x,0)-y_{0}(x) \bigr\Vert ^{2}_{L^{2}(\Omega )} \\ &\quad \quad {} +\delta \int_{0}^{T} \Vert \zeta \Vert _{H^{2}(\Omega )}^{2}\,dt+\delta \bigl\Vert \zeta (x,0) \bigr\Vert _{L^{2}(\Omega )}^{2} \\ &\quad \leq C(\delta )\sum_{6}^{10}\hat{\eta }_{i}^{2}+\delta \bigl\Vert Y _{N}-y(U_{N}) \bigr\Vert ^{2}_{L^{2}(0,T;L^{2}(\Omega ))}. \end{aligned} \end{aligned}$$

Hence, let *δ* be small enough, by simplifying both sides, we have

4.14 $$ \bigl\Vert y(U_{N})-Y_{N} \bigr\Vert ^{2}_{L^{2}(0,T;L^{2}(\Omega ))}\leq C\sum_{6}^{10} \hat{\eta }_{i}^{2}. $$

Then (4.3) follows from (4.13) and (4.14). □

From Theorem 3.1 and Theorem 4.1, we have the following a posteriori error estimate.

### Theorem 4.2

*Let* $(y,p,u)$ *and* $(Y_{N},P_{N},U_{N})$ *be the solutions of* (2.8)*–*(2.10) *and* (2.25)*–*(2.29), *respectively*. *Assume that all the conditions in Theorem *3.1 *and Lemma *4.1 *are valid*. *Then*

4.15 $$ \begin{aligned}[b] & \Vert Y_{N}-y \Vert _{L^{2}(0,T;L^{2}(\Omega ))}^{2}+ \Vert P_{N}-p \Vert _{L^{2}(0,T;L ^{2}(\Omega ))}^{2}+ \Vert U_{N}-u \Vert _{L^{2}(0,T;L^{2}(\Omega ))}^{2}\\&\quad \leq C \eta_{1}^{2}+C\sum_{i=2}^{10} \hat{\eta }_{i}^{2},\end{aligned} $$

*where* $\eta_{1}^{2}$, $\hat{\eta }_{i}^{2}$, $i=2,\ldots,10$ *are defined in Theorem *3.1 *and Theorem *4.1.

### Proof

From Theorem 3.1 and Theorem 4.1, we have

4.16 $$\begin{aligned} \begin{aligned}[b] \Vert u-U_{N} \Vert _{L^{2}(0,T;L^{2}(\Omega ))}^{2}&\leq C\eta_{1}^{2}+C \bigl\Vert \tilde{P}_{N}-p(U_{N}) \bigr\Vert _{L^{2}(0,T;L^{2}(\Omega ))}^{2} \\ &\leq C \eta_{1}^{2}+C \Vert \tilde{P}_{N}-P_{N} \Vert _{L^{2}(0,T;L^{2}(\Omega ))}^{2} \\ &\quad {} +C \bigl\Vert P_{N}-p(U_{N}) \bigr\Vert _{L^{2}(0,T;L^{2}(\Omega ))}^{2} \\ &\leq C \eta_{1}^{2}+C\sum_{i=2}^{10} \hat{\eta }_{i}^{2}+C \Vert \tilde{P} _{N}-P_{N} \Vert _{L^{2}(0,T;L^{2}(\Omega ))}^{2}. \end{aligned} \end{aligned}$$

By virtue of *A* being positive definite, by using the Poincaré inequality, we can get

4.17 $$ \Vert \tilde{P}_{N}-P_{N} \Vert _{L^{2}(0,T;L^{2}(\Omega ))}^{2}\leq C \int_{0} ^{T} \int_{\Omega } \bigl\vert A^{*}\nabla ( \tilde{P}_{N}-P_{N}) \bigr\vert ^{2}\,dx\,dt=C \hat{ \eta }_{3}^{2}. $$

Then it follows from (4.16) and (4.17) that

4.18 $$ \Vert u-U_{N} \Vert _{L^{2}(0,T;L^{2}(\Omega ))}^{2} \leq C\eta_{1}^{2}+C\sum_{i=2}^{10} \hat{\eta }_{i}^{2}. $$

Note that

4.19 $$\begin{aligned}& \Vert Y_{N}-y \Vert _{L^{2}(0,T;L^{2}(\Omega ))}^{2} \leq \bigl\Vert Y_{N}-y(U_{N}) \bigr\Vert _{L^{2}(0,T;L^{2}(\Omega ))}^{2} + \bigl\Vert y(U_{N})-y \bigr\Vert _{L^{2}(0,T;L^{2}( \Omega ))}^{2}, \end{aligned}$$

4.20 $$\begin{aligned}& \Vert P_{N}-p \Vert _{L^{2}(0,T;L^{2}(\Omega ))}^{2} \leq \bigl\Vert P_{N}-p(U_{N}) \bigr\Vert _{L^{2}(0,T;L^{2}(\Omega ))}^{2} + \bigl\Vert p(U_{N})-p \bigr\Vert _{L^{2}(0,T;L^{2}( \Omega ))}^{2}, \end{aligned}$$

and

4.21 $$\begin{aligned}& \bigl\Vert y(U_{N})-y \bigr\Vert _{L^{2}(0,T;L^{2}(\Omega ))}^{2}\leq C \Vert u-U_{N} \Vert _{L ^{2}(0,T;L^{2}(\Omega ))}^{2}, \end{aligned}$$

4.22 $$\begin{aligned}& \bigl\Vert p(U_{N})-p \bigr\Vert _{L^{2}(0,T;L^{2}(\Omega ))}^{2}\leq \bigl\Vert y(U_{N})-y \bigr\Vert _{L ^{2}(0,T;L^{2}(\Omega ))}^{2}\leq C \Vert u-U_{N} \Vert _{L^{2}(0,T;L^{2}( \Omega ))}^{2}. \end{aligned}$$

Therefore, (4.15) follows from (4.16) and (4.18)–(4.22). □

## Concluding remarks and future work

In this paper, we present a fully discrete scheme in which we use the backward Euler scheme in time and use the spectral approximation in space for the nonlinear parabolic optimal control problem. By using the orthogonal projection operator and some auxiliary equations, we obtain $L^{2}(H^{1})-L^{2}(L^{2})$ a posteriori error estimates of the spectral approximation solutions for both the state and the control, and also obtained $L^{2}(L^{2})-L^{2}(L^{2})$ a posteriori error estimates of the spectral approximation solutions for both the state and the control.

The results obtained and techniques used can be extended to such control problems with more general objective functions. Furthermore, we shall consider the spectral approximation for hyperbolic optimal control problems.
